# Supplementary material for: Human CD4+ T-Cell Clone Expansion Leads to the Expression of the Cysteine Peptidase Inhibitor Cystatin F
Source: Int J Mol Sci. 2021 Aug 5;22(16):8408. doi: 10.3390/ijms22168408 (PMC8395124; doi:10.3390/ijms22168408)
Supplement: Supplementary file 1 [file ijms-22-08408-s001.zip › ijms-1257639-supplementary.pdf]

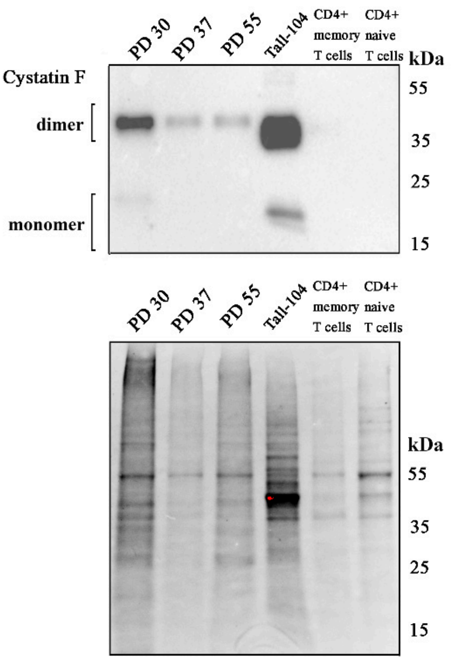

Supplementary Figure S1. Cystatin F is expressed in long-term cultured CD4+ T cells but not in naïve and memory CD4+ T cells. (**A**) Western blot analysis and cystatin F immunodetection in cell lysates of CD4+ T-cell clones, Tall-104 cell line, human memory (CD45RA-) CD4+ T cells and human naïve (CD45RA+) CD4+ T cells. Untouched naive CD4+ T helper cells (CD4+CD45RA+) were isolated from PBMCs of healthy donor using the Naive CD4+ T Cell Isolation Kit II, an LS Column, and a MiniMACS™ Separator (all from Miltenyi Biotech). Untouched CD4+ memory T cells were isolated from human PBMCs using the CD4+ Central Memory T Cell Isolation Kit, an LD Column, and a MiniMACS™ Separator (Miltenyi Biotec). Imaging of stain-free activated protein membrane was used to show protein loading.

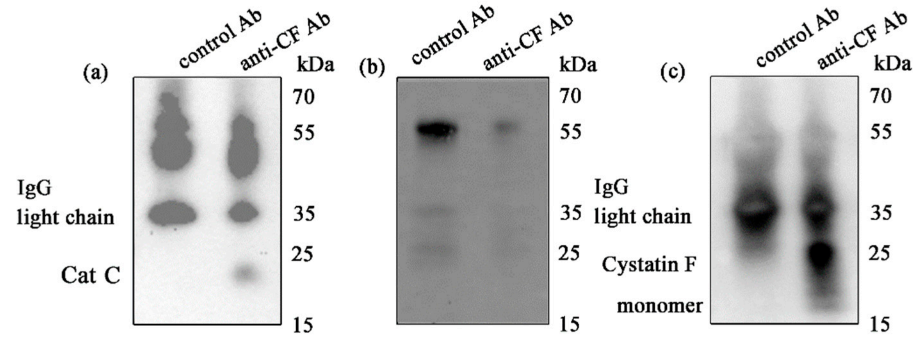

Supplementary Figure S2. One of the main targets of cystatin F in long-term cultured CD4+ T cells is cathepsin C. **(a)** Western blot analysis and Cat C immunodetection in immunoprecipitates with anti-cystatin F antibodies and negative control antibodies against lectin isolated from *Macrolepiota procera* for interaction of cystatin F with Cat H. (**b**) Imaging of stain-free activated protein membrane was used to confirm equal protein loading. (**c**) Western blot analysis and cystatin F immunodetection in immunoprecipitates with anti-cystatin F antibodies and negative control antibodies against lectin isolated from *Macrolepiota procera.*

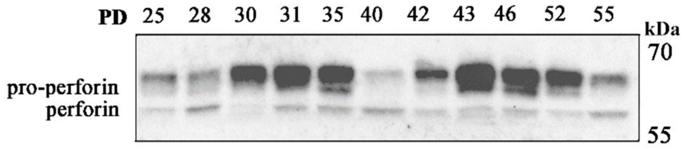

Supplementary Figure S3. Long-term cultured CD4+ T cells express pro and mature form of perforin. Western blot analysis and perforin immunodetection in cell lysates of CD4+ T cell clones. Pro form of perforin and mature perforin are indicated on the left. In order to confirm the presence of mature form of perforin the lysates were resolved by Novex™ WedgeWell™ 6%, Tris-Glycine under nonreducing conditions. The migration of molecular weight standards is indicated.

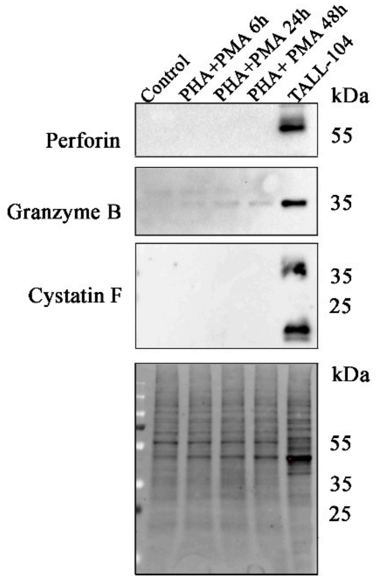

Supplementary Figure S4. PMA and PHA stimulated Jurkat cells expess granzyme B but not cystatin F. Western blot analysis and perforin, granzyme B and cystatin F immunodetection in cell lysates of Jurkat cells treated with phorbol 12-myristate 13-acetate (PMA) and the T-cell mitogen phytohemagglutinin (PHA) for 6, 24, and 48 h. Tall-104 cells were used as positive control. Imaging of stain-free activated protein membrane was used to show protein loading.
